# Comments on: “Mesenchymal stem cells transplantation for perianal fistulas: a systematic review and meta-analysis of clinical trials”

**DOI:** 10.1186/s13287-023-03607-x

**Published:** 2023-12-18

**Authors:** Fang Cheng

**Affiliations:** Division of Gastroenterology, Zigong First People’s Hospital, 42 Shangyihao Road, Zigong, 643000 Sichuan Province China

**Keywords:** Perianal fistulas, Mesenchymal stem cells, Systematic review and meta-analysis

## Abstract

The meta-analysis by Wang et al. (Stem Cell Res Ther 14(1):103, 2023) aims to explore whether mesenchymal stem cells are effective for perianal fistulas. The authors indicated that the difference in cell types, cell sources and cell dosages did not influence mesenchymal stem cells’ efficacy, which may not be accurate. I think that local treatment with higher dosages of mesenchymal stem cells seems to not result in a higher healing rate. And, future trials should focus on donor characteristics considering past medical history of further autoimmunity, timely and cost-effective treatment to lighten the optimized therapeutic goals. In the future, it will be interesting to assess the safety and feasibility of injection of fibrin glue combined with mesenchymal stem cells in perianal fistulas.

## Introduction

I read with great interest the meta-analysis by Wang et al. [1] reporting the mesenchymal stem cells (MSCs) transplantation for perianal fistulas (PFs). I appreciate the authors’ hard work. However, I have several concerns about the study. First, the meta-analyses aimed to explore different dosage of MSCs for the treatment of complex PFs. The pooled analysis showed that low-dosage MSCs subgroup (RR = 1.51, 95% CI 1.02, 2.21; *P* = 0.04) and high-dosage MSCs subgroup (RR = 1.30, 95% CI 1.02, 1.66; *P* = 0.03) can obtain higher healing rates (HR) than the control group. Based on the results of this meta-analysis, the authors indicated that there was no difference of treatment efficacy concerning dosage change (< 10 × 10^7^ vs ≥ 10 × 10^7^). I think this conclusion is not accurate. There is no direct comparison between different doses of MSCs. Therefore, this meta-analysis came to the conclusion that MSC therapy is effective, but it could not indicate whether factors of dosage influence treatment efficacy. Molendijk et al. [2] reported that administration of 3 × 10^7^ MSCs resulted in higher fistula healing compared with 9 × 10^7^ MSCs treatment. There was also a clinical trial indicated that patients who received 20 million cells were found to have significantly greater LVEF and showed a reduction in scar size in comparison with those who received 200 million MSCs [3]. So, these evidences supported that local treatment with higher dosages of MSCs seems to not result in a higher HR. Higher cell concentrations could result in a lower survival rate and/or cell function, and secondly, a larger number of cells could behave immunogenically resulting in increased clearance or deactivation of the cells [4]. Second, the meta-analyses aimed to explore different sources of MSCs for the treatment of PFs. In this meta-analyses, autologous and allogenic MSCs could both improve HR compared with control (55.65% versus 45.68%; 56.78% versus 38.32%). This meta-analysis concluded that autologous MSCs were effective for fistulas as well as allogenic MSCs. I agree with this argument. But, in the short conclusion part of the abstract that the authors indicated the difference cell sources did not influence MSCs’ efficacy. I think this conclusion is not accurate. There is no direct comparison between different sources of MSCs. And disease-related effects on autologous MSCs must be taken into account. There was an in vitro assessment reported decreased immunosuppressive function of MSCs derived from CD patient vs healthy donors, prioritizing allogenic transplant over autologous [5]. And, we should know autologous MSCs are not immediately available upon request because isolation and expansion of MSCs to sufficient numbers of cells require weeks, resulting in treatment delay. Therefore, we think future trials should focus on donor characteristics considering past medical history of further autoimmunity, timely and cost-effective treatment to lighten the optimized therapeutic goals. Finally, cell therapy strategies using MSCs carried in fibrin glue (FG) have shown promising results in regenerative medicine [6, 7]. MSCs highlighted as potential candidates due to their angiogenic, anti-apoptotic and immunomodulatory properties, in addition to their ability to differentiate into several specialized cell lines. Cells can be carried through fibrin glue, which acts as a temporary matrix that favors cell–matrix interactions and allows local and paracrine functions of MSCs. In this meta-analysis, some included studies reported efficacy of local FG combined with MSCs therapy for PF. Whether there was any promotion effect of the MSC plus fibrin glue therapy remains unknown. Hence, it will be interesting to assess the safety and feasibility of injection of FG combined with MSCs in PFs.

## Conclusions

In this view, in respect of cellular dosage, only controlled trials with strict comparison between different dosage of MSCs can determine the suitability cell to treat PFs. And, higher dosages of MSCs seem to not result in a higher HR. Moreover, future trials should focus on donor characteristics considering past medical history of further autoimmunity, timely and cost-effective treatment to lighten the optimized therapeutic goals. And, the combination of FG with MSCs could be studied in order to elucidate the possibility of synergistic or additive effects.

## Data Availability

Not applicable.

## Abbreviations

MSCs: Mesenchymal stem cells
PF: Perianal fistulas
HR: Healing rates
FG: Fibrin glue
